# NLK Is a Novel Therapeutic Target for PTEN Deficient Tumour Cells

**DOI:** 10.1371/journal.pone.0047249

**Published:** 2012-10-29

**Authors:** Ana M. Mendes-Pereira, Christopher J. Lord, Alan Ashworth

**Affiliations:** 1 The Breakthrough Breast Cancer Research Centre, The Institute of Cancer Research, London, United Kingdom; 2 Cancer Research UK Gene Function Group, The Institute of Cancer Research, London, United Kingdom; University Magna Graecia, Italy

## Abstract

*PTEN* (*Phosphatase and tensin homolog*) is a tumour suppressor gene commonly defective in human cancer, and is thus a potentially important therapeutic target. Targeting tumour suppressor loss-of-function is possible by exploiting the genetic concept of synthetic lethality (SL). By combining the use of isogenic models of PTEN deficiency with high-throughput RNA interference (RNAi) screening, we have identified *Nemo-Like Kinase* (*NLK*) inhibition as being synthetically lethal with PTEN deficiency. This SL is likely mediated by the transcription factor FOXO1 (Forkhead box O1), an NLK substrate, as the selectivity of *NLK* gene silencing for PTEN deficient cells can be reversed by *FOXO1* knockdown. In addition, we provide evidence that PTEN defective cells targeted by *NLK* gene depletion undergo senescence, suggesting that NLK function is critical for the continued proliferation of PTEN deficient cells. Taken together, these data provide new insight into the potential of targeting of NLK to treat a range of tumourigenic conditions characterised by PTEN deficiency.

## Introduction

Loss of function mutations in the tumour suppressor gene *PTEN* or lack of PTEN expression have both been observed in a wide range of human tumours [1]. *PTEN* encodes a phosphatase whose activity antagonises PI3 kinase signaling by dephosphorylating the plasma membrane lipid phosphoinositide-3,4,5-trisphosphate (PIP3). The loss of PTEN phosphatase activity is thought to foster tumourigenesis, at least in part, by causing downstream constitutive activation of AKT. In addition to this role, PTEN has been suggested to have a nuclear function in maintaining genomic stability [2].

The presence of tumour-specific loss-of-function mutations in *PTEN* raises the possibility of identifying PTEN synthetic lethal interactions that could be exploited therapeutically. Previously, we have shown that PTEN deficiency is synthetically lethal with inhibition of PARP1 [3], and effect predicted from the proposed role of PTEN in maintaining genomic stability [2]. It is likely that additional SLs involving PTEN exist, and these might prove valuable candidate therapeutic approaches. High-throughput genetic screening using RNA interference (RNAi) represents a straightforward method to identifying SLs in a relatively unbiased fashion [4].

## Results and Discussion

**Figure 1:**
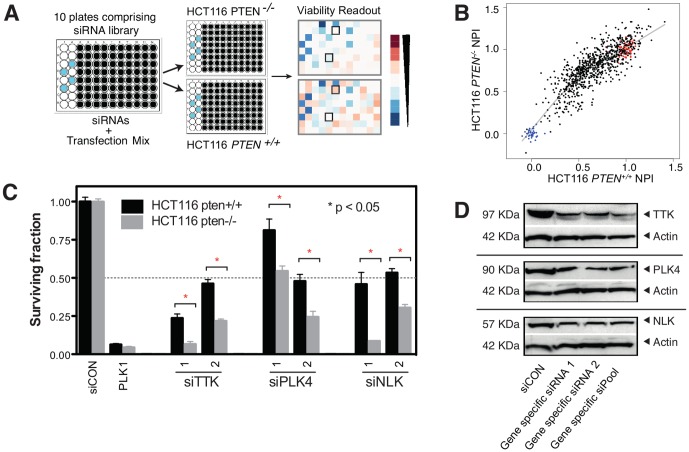
PTEN synthetic lethality screening. (**A**) High-throughput screen (HTS) schematic. PTEN proficient and deficient HCT116 cells (Horizon Discovery) were siRNA screened as described in the Methods. Example heatmaps from luminescence measurements in 96 well plates are shown. For screening, a siGENOME SMARTPool library (Dharmacon) targeting 779 kinases and kinase-related genes was used. (**B**) Results from data analysis of combined triplicate screens, represented as a scatter plot of Normalised Percent Inhibition (NPI) values from HCT116 *PTEN^−/−^* and HCT116 *PTEN^+/+^* screens. Blue dots corresponded to siPLK effects and red dots corresponded to siCON negative control effects. NPI values below the trend line shown were considered as candidate synthetic lethalities. (**C**) Validation of PTEN synthetic lethal hits from the HTS using multiple siRNAs for each gene. Cells were transfected with siRNA as per the HTS and surviving fractions calculated from CellTiterGlo luminescence measurements five days later. Surviving fraction data from HCT116 *PTEN^−/−^* and HCT116 *PTEN^+/+^* cells transfected with each siRNA are shown. (**D**) Western blot showing silencing effects of each siRNA.

We used a high-throughput screening (HTS) approach to identify novel SLs involving PTEN (Figure 1A). Specifically, we reverse-transfected PTEN wild type and null human tumour cell lines with a short-interfering (si)RNA library arrayed in 96-well plates and measured effects of each siRNA on cell proliferation using CellTiter-Glo*®* reagent (Promega) five days after transfection. The siRNA library we used comprised 779 siRNA SMART-pools (Dharmacon), each designed to target a specific kinase or kinase-related gene. We selected this gene subset to screen given its inherent pharmacological tractability. To maximise the possibility of identifying SLs specific to PTEN, we carried out triplicate screens in a pair of isogenically matched HCT116-derived wild-type (*PTEN^+/+^*) and *PTEN* ^−/−^ colorectal tumour cell lines [5]. PTEN deficiency in this model was achieved by targeting a truncating mutation to both copies of *PTEN* at exon 2, with the resultant mutant alleles encoding an ostensibly dysfunctional PTEN mutant protein consisting of only the 24 N-terminal amino-acids [6], [7].

The sensitivity of the screen and its overall performance were monitored through a series of commonly used HTS metrics [8]. These included: (i) PLK1 siRNA causing a reduction in viability of more than 90% in both cell types, when compared to transfection with a non-targeting siRNA control (siCON); (ii) Z′-factor estimation between negative (siCON) and positive (siPLK1) control wells where a Z′≥0.5 was set as a threshold of acceptable dynamic range (Figure S1); and (iii) <10% inhibition in cell proliferation caused by control non-targeting siRNA compared to mock transfected wells. Transfected cell numbers were titrated to ensure equal transfection efficiency and confluency in PTEN null and wild-type cells. Following screen optimisation, each cell line was screened three times and the data from replica screens combined in the final analysis (Figure 1B).

To identify PTEN selective effects we first calculated NPI (normalised percent inhibition) scores [9] for each siRNA pool in both PTEN^+/+^ and PTEN^−/−^ cell lines. To calculate NPI scores, which scale cell viability effects according to maximal and minimal effects, we defined the maximal inhibitory effect as that caused by siRNAs targeting PLK1 and the minimal effect as that caused by non-targeting control siRNAs. This procedure equalised data distributions between both lineages, allowing the comparison of NPI scores between *PTEN* ^−/−^ and *PTEN^+/+^* cells. This ultimately allowed each siRNA pool to be ranked ordered according to its *PTEN* ^−/−^ selective effect.

We selected the 20 greatest *PTEN* ^−/−^ selective effects (Table S1) for technical validation using the original screening protocol in a lower-throughput format to minimise false positive effects. This analysis highlighted three genes with clear PTEN selective effects: *NLK* (*Nemo-Like Kinase*), *PLK4* (*Polo-Like Kinase 4*), and *TTK* (*MPS1, MonoPolar Spindle 1*) (Figure 1C). Recently, we have used functional viability profiling in non-isogenic breast tumour cell line models to identify candidate SL effects, an analysis that identified TTK as synthetically lethal with PTEN [10]. The identification of the PTEN/TTK SL in the screen described here indicated that the HTS performed on the isogenic models was able to identify *bona fide* effects. The NLK, PLK4 and TTK effects were then subject to further revalidation to exclude possible RNAi off-target effects. Given that a phenotype caused by two distinct siRNA species suggests a lower likelihood of an off-target effect [11], we reassayed viability effects using each of the four different siRNA species that comprise the SMARTpools used in the primary screen. Analysis using these “deconvoluted” pools resulted in the identification of at least two siRNAs for each gene that reproduced the original effects (Figure 1C), In addition, the ability of these individual siRNAs to silence their target genes was confirmed by immunoblotting (Figure 1D).

**Figure 2:**
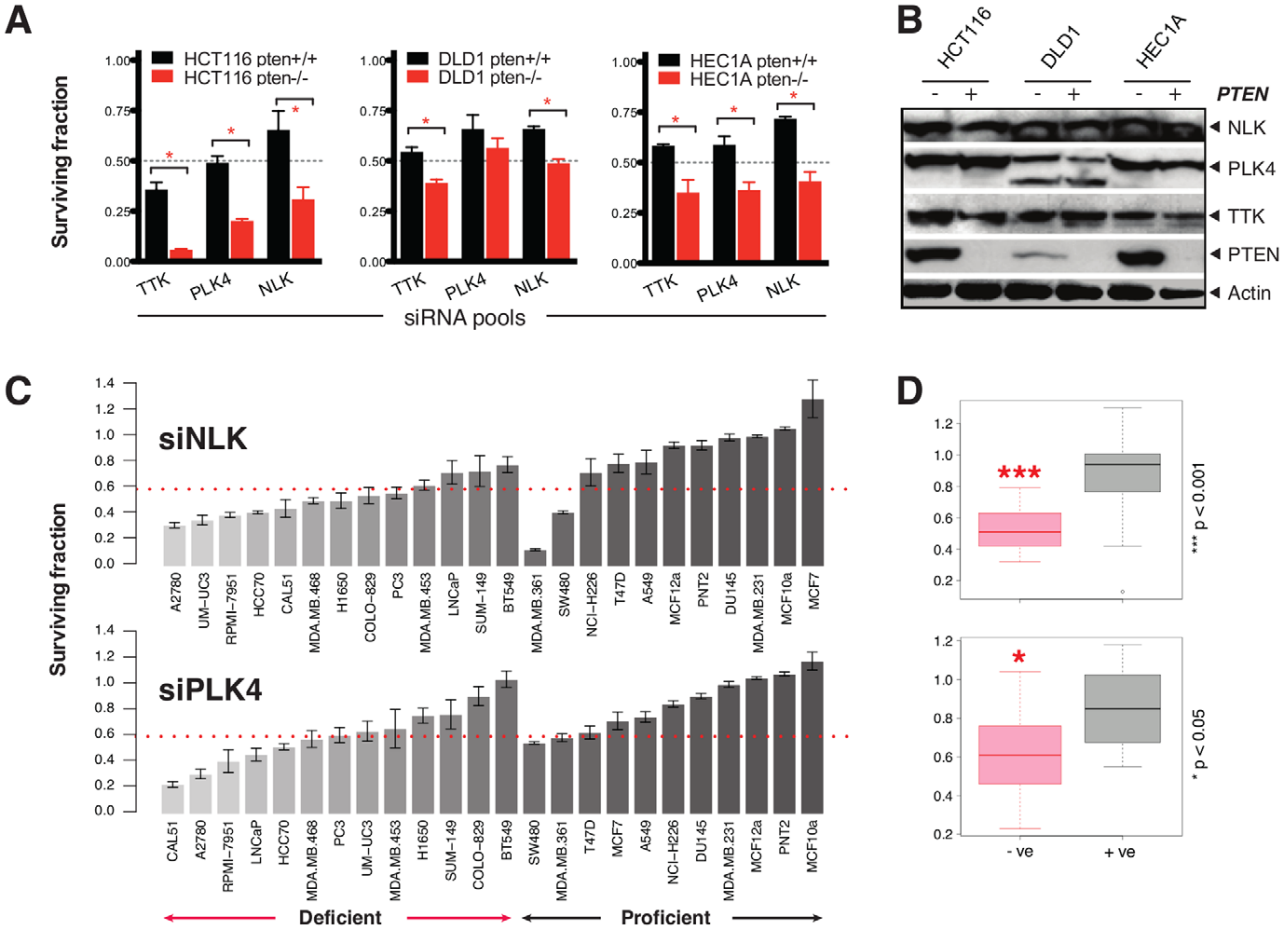
Validation of the PTEN/NLK synthetic lethality. (**A**) Validation of PTEN synthetic lethal hits in additional isogenic models. Isogenic HCT116, DLD1 and HEC1A PTEN proficient and deficient models were transfected with siRNA as in Figure 1 and surviving fractions calculated five days later. Surviving fraction data from HCT116, HEC1A and DLD1 models is shown. * *p* value<0.05 Student's *t* test (**B**) Western blot showing expression of each candidate gene in three PTEN isogenic models. (**C**) Cell inhibitory effect of siRNA targeting NLK or PLK4 in a panel of 24 human tumour cell lines (11 PTEN proficient models and 13 PTEN deficient models – see Table 1). (**D**) Box plots illustrating surviving fraction data for PTEN proficient and deficient groups shown in (C). *p*-values were calculated with Student's *t*-test.

Highly penetrant “hard” SLs that are relatively unaffected by additional genetic modifications are preferred over those that are readily abrogated by other changes in the genetic background of a cell. It seems likely that such hard synthetic lethalities are more likely to be robust in genetically heterogeneous tumours [12]. To address this issue, we used two additional isogenic models of PTEN deficiency derived from (i) HEC1A endometrial and (ii) DLD1 colorectal tumour cell lines. In both HEC1A and DLD1 models, homozygous mutation of *PTEN* was achieved using the same targeting strategy as for the HCT116 models, where truncating mutations were introduced into both copies of *PTEN* exon 2. Both, NLK and PLK4 SLs re-validated in the HEC1A model, whilst the PTEN/PLK4 SL failed to re-validate in the DLD1 isogenic matched pair (Figure 2A), possibly due to reduced basal levels of PLK4 in DLD1 cells (Figure 2B). Although the PTEN/PLK4 SL seemed not as prevalent as the PTEN/NLK SL, it is possible that in some PTEN null cell types, PLK4 inhibition exploits the underlying centromeric dysfunction of PTEN mutant tumor cells. Aneuploidy is frequently observed in both human breast carcinomas with low expression of PTEN and prostatic intraepithelial neoplasia from *Pten* mutant mice. As PLK4 is required for normal spindle function, controlled mitotic exit and the suppression of chromosomal aneuploidy, it is possible that PLK4 inhibition further exacerbates aneuploidy in PTEN null cells and ultimately drives cell death.

To further assess the generality of our observations, we examined NLK and PLK4 SLs in a wide panel of genetically heterogenous PTEN deficient or proficient tumour cell lines (Table 1). Here we used a combination of two validated siRNAs to target each gene, alongside appropriate positive (siPLK1) and negative (siCON) controls (Figure S2). Silencing of either NLK or PLK4, had a greater cell inhibitory effect in the PTEN deficient cohort when compared to the PTEN proficient group (Figure 2C, 2D), an effect especially evident for NLK silencing. Nevertheless, the extent of the PTEN/NLK SL did vary across the cell line panel and it seems possible that other genetic differences between tumour cell models modulate the penetrance of the PTEN/NLK SL.

**Table 1:** List of 24 cell lines, categorised as PTEN proficient (n = 11) or deficient (n = 13) as defined by western blotting.

Cell line	Origin
**PTEN proficient models**	
MDA-MB-231	Breast
MDA-MB-361	Breast
MCF7	Breast
MCF10A	Breast
MCF12A	Breast
T47D	Breast
A549	Lung
NCI-H226	Lung
PNT2	Prostate
DU145	Prostate
SW480	Colon
**PTEN deficient models**	
MDA-MB-453	Breast
MDA-MB-468	Breast
SUM149	Breast
HCC70	Breast
CAL51	Breast
BT549	Breast
COLO-829	Melanoma
RPMI-7951	Melanoma
PC3	Prostate
LNCaP	Prostate
UM-UC3	Bladder
H1650	Lung
A2780	Ovary

Given the generality of the NLK/PTEN SL, we went on to investigate the mechanism of NLK action in the context of PTEN deficiency. Opposing the pro-oncogenic effects of PTEN dysfunction are molecular networks that normally promote quiescence, senescence or cell death. These include the Forkhead O-class transcription factors such as FOXO1 (‘Forkhead-box-O1’ isoform). In the face of PTEN deficiency, FOXO1 can exert tumour suppressive effects by mediating the transcription of genes such as *p21*, *p27*, and *BIM* that promote quiescence, senescence or cell death. However, this tumour suppressive function of FOXO1 can in turn be circumvented by the AKT activation that characterises PTEN deficiency. The phosphorylation of FOXO1 by activated AKT causes FOXO1 to be excluded from the nucleus and ultimately degraded in the cytoplasm, minimizing its ability to drive a tumour suppressive transcriptional programme [13].

**Figure 3:**
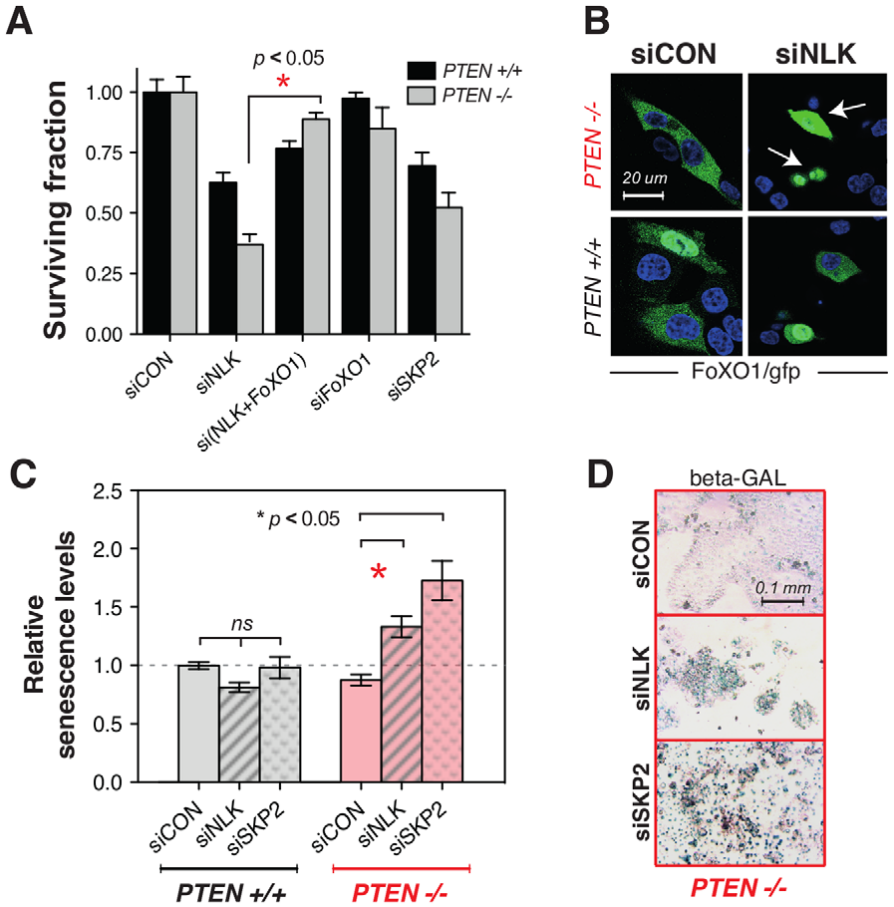
PTEN/NLK synthetic lethality is abrogated by FOXO1 silencing. (**A**) Survival analysis of HCT116 cells transfected with NLK and/or FOXO1 siRNA. HCT116 *PTEN^−/−^* and HCT116 *PTEN^+/+^* cells were transfected with siRNA targeting NLK and FOXO1 as shown and surviving fractions determined after five days. The *p* value (*) was calculated using Student's t test. (**B**) Nuclear localisation of FOXO1 is enhanced in PTEN deficient cells upon NLK silencing. HCT116 isogenic cells were co-transfected with GFP-tagged *FOXO1* cDNA in addition to control (non targeting) siRNA (siCON) or NLK siRNA. Two days later cells were fixed and stained with DAPI. Green signal represents GFP-FOXO1 and blue signal represents nuclear DAPI (nuclear) staining. Arrows indicate cells with nuclear localisation of FOXO1. (**C**) Senescence is increased by NLK siRNA in PTEN deficient cells. Bar chart of relative relative senescence levels caused by NLK silencing are shown. HCT116-derived PTEN isogenic cell lines were reversed transfected with a pool of two validated siRNAs against NLK, as well as siCON pool#2 (Dharmacon) as non-targeting control, using RNAiMAX (Invitrogen). Seven days after transfection cells were fixed and incubated overnight at 37°C in a solution containing X-gal. (**D**) Representative images for β-Galactosidase staining of PTEN deficient cells. Blue staining indicates β-Galactosidase.

One of the established functions of NLK is the AKT-independent phosphorylation of FOXO1; this phosphorylation also causes FOXO1 inactivation via its nuclear exclusion [14]. We hypothesised that NLK inhibition could be synthetically lethal with PTEN deficiency as it causes reactivation of FOXO1 and therefore cellular senescence. To test if the PTEN/NLK synthetic lethality was FOXO1 dependent, we transfected PTEN null cells with both NLK and FOXO1 siRNA and assessed the effect on cell inhibition. Whilst FOXO1 siRNA alone did not selectively target PTEN null cells and NLK1 siRNA caused PTEN synthetic lethality (as before), FOXO1 siRNA reversed the PTEN/NLK synthetic lethality (Figure 3A, Figure S3), supporting the hypothesis that the PTEN/NLK SL is FOXO1 dependent. NLK silencing also caused an increase in nuclear FOXO1 localisation (Figure 3B), again supporting the hypothesis. Furthermore, NLK silencing induced senescence in PTEN deficient cells when compared to PTEN proficient counterparts (Figure 3C, 3D), suggesting that the instigation of a senescent programme by FOXO1 could explain the PTEN/NLK synthetic lethality.

Our study demonstrates that NLK silencing inhibits PTEN deficient tumour cells. Our mechanistic dissection of this effect suggests that re-instatement of a FOXO1-driven process, possibly cellular senescence that normally suppresses oncogenesis, could explain the PTEN selective effect of NLK inhibition. As a potential route to identifying leads for cancer drug discovery, others have already identified compounds that cause the translocation FOXO1 to the nucleus [15], [16]. The data presented here suggests that inhibition of NLK could provide a similar target for drug development and the generation of toolbox small molecule NLK inhibitors would allow proof of concept studies to be performed including an *in vivo* assessment of the PTEN/NLK SL. Ultimately, PTEN deficiency could be used as a biomarker to identify patients likely to respond to a clinical NLK inhibitor.

## Methods

### PTEN synthetic lethality screening

PTEN proficient and deficient HCT116 cells (Horizon Discovery) were maintained in McCoy's 5A (Invitrogen) supplemented with 10% (v/v) foetal calf serum (FBS), glutamine and antibiotics and reverse transfected with a siRNA library (final siRNA concentration per well, 50 nM) using RNAiMAX (Invitrogen). For screening, a siGENOME SMARTPool library (Dharmacon) targeting 779 kinases and kinase-related genes was used. The following day media was changed and cells cultured for five subsequent days, at which point cell viability was assessed using CellTiterGlo Luminescent Cell Viability Assay (Promega). For validation experiments, cells were transfected with siRNA as per the HTS and surviving fractions calculated from CellTiterGlo luminescence measurements five days later. All other cell lines were sourced from ATCC and maintained as per the supplier's instructions.

### Western blots

Cells were transfected with siRNA and cell lysates generated 48 hours later. Whole-cell protein extracts were prepared from cells lysed with RIPA buffer (Upstate) supplemented with protease inhibitor cocktail tablets (Roche). Protein concentrations were measured using Bio-rad Protein Assay Reagent. For western blot analysis, lysates were electrophoresed on Novex 4–12% gradient bis-tris pre-cast gels (Invitrogen) and immunoblotted overnight at 4°C with antibodies targeting the following epitopes: NLK (C-20: sc-8210, Santa Cruz Biotechnology), PLK4 (#3258, Cell Signaling), TTK (C-19: sc-540, Santa Cruz Biotechnology) and β-Actin (I19: sc-1616, Santa Cruz Biotechnology). Incubation with primary antibody was followed by incubation with a horseradish peroxidase-conjugated secondary antibody and chemi-luminescent detection of proteins (Amersham Pharmacia).

### FOXO1 detection

HCT116 isogenic cells were co-transfected with GFP-tagged *FOXO1* cDNA (Origene) in addition to control (non targeting) siRNA (siCON) or NLK siRNA. Two days later cells were fixed in 4% (w/v) para-formaldehyde, after which nuclei were stained with 4′,6-diamidino-2-phenylindole (DAPI; D21490, Invitrogen). DAPI and GFP signals from cells were captured using an inverted confocal Zeiss LSM710 microscope.

### Assessment of senescence

HCT116-derived PTEN isogenic cell lines were reversed transfected with a pool of two validated siRNAs against NLK, as well as siCON pool#2 (Dharmacon) as non-targeting control, using RNAiMAX (Invitrogen). Seven days after transfection cells were fixed in PBS containing 2% (w/v) formaldehyde and 0.2% (v/v) glutaraldehyde, rinsed and incubated overnight at 37°C in a solution containing X-gal according to manufacturer instructions (Senescence β-Galactosidase Staining Kit, Cell Signaling Technology). After aspirating staining solution, cells were overlaid with 70% (v/v) glycerol and stored at 4°C until reading absorbance at 595 nm (A_595_) in a Victor X5 plate reader (Perkin Elmer). A_595_ values were normalised to the A_595_ signal in PTEN proficient cells transfected with siCONpool#2 transfected cells to give relative senescence levels in each experimental sample.

## Supporting Information

Figure S1 siRNA Screening Quality-Metrics. (A) Quality control (QC) illustrating behavior of controls, with respective Z′-factors, for a representative screen replicate covering the ten 96-well plates covering the siRNA library. (B) Boxplots showing distribution of the cell inhibitory effects of 779 siRNAs, averaged from three screen replicates, for both PTEN deficient and proficient HCT116-derived lineages. “Neg” represents the effect of non-targeting control siRNA and “pos” the effect of siRNA targeting PLK1. Negative Z scores represent cell inhibitory effects.
(PDF)

Figure S2 Evaluation of control siRNAs (siCON and siPLK1) viability effects in a panel of 24 tumour cell lines. “Deficient” and “Proficient” indicate PTEN status.
(PDF)

Figure S3 Confirmation of silencing by FOXO1 and SKP2 siRNAs in HCT116 cells. Antibodies targeting epitopes of FOXO1 (C29H4, cell signaling #2880) and SKP2 (cell signaling #4358) were used for immuno-blotting.
(PDF)

Table S1 List of synthetic candidate hits from siKinome isogenic screen highlighting Nemo-like kinase (NLK) results.
(PDF)
