# Supplementary material for: NLK Is a Novel Therapeutic Target for PTEN Deficient Tumour Cells
Source: PLoS One. 2012 Oct 29;7(10):e47249. doi: 10.1371/journal.pone.0047249 (PMC3483146; doi:10.1371/journal.pone.0047249)
Supplement: Figure S1 — siRNA Screening Quality-Metrics. (A) Quality control (QC) illustrating behavior of controls, with respective Z′-factors, for a representative screen replicate covering the ten 96-well plates covering the siRNA library. (B) Boxplots showing distribution of the cell inhibitory effects of 779 siRNAs, averaged from three screen replicates, for both PTEN deficient and proficient HCT116-derived lineages. “Neg” represents the effect of non-targeting control siRNA and “pos” the effect of siRNA targeting PLK1. Negative Z scores represent cell inhibitory effects. (PDF) [file pone.0047249.s001.pdf]

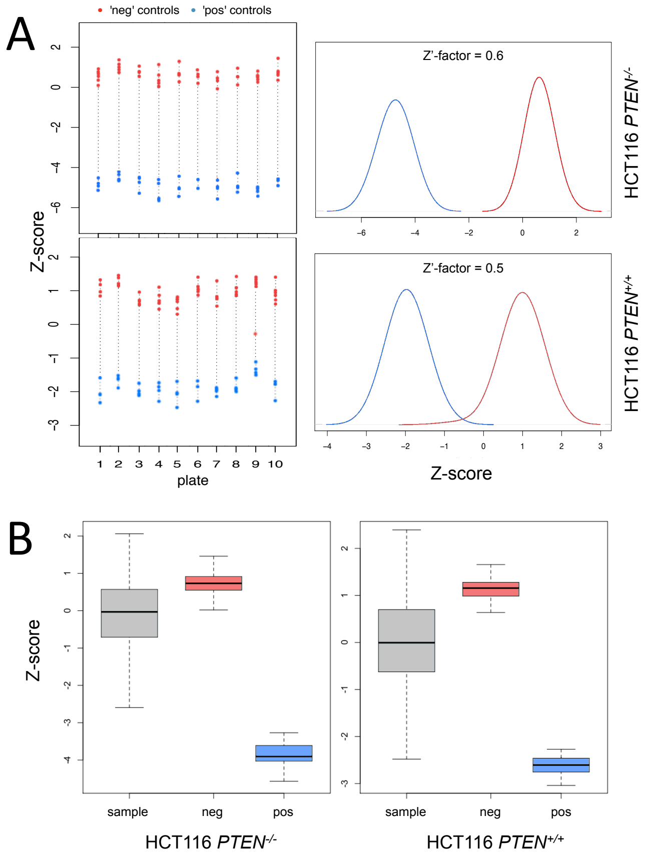


**Figure S1. siRNA Screening Quality-Metrics**

**(A)** Quality control (QC) illustrating behavior of controls, with respective Z'-factors, for a representative screen replicate covering the ten 96-well plates covering the siRNA library. **(B)** Boxplots showing distribution of the cell inhibitory effects of 779 siRNAs, averaged from three screen replicates, for both PTEN deficient and proficient HCT116-derived lineages. "Neg" represents the effect of non-targeting control siRNA and "pos" the effect of siRNA targeting PLK1. Negative Z scores represent cell inhibitory effects.
