# Supplementary material for: NLK Is a Novel Therapeutic Target for PTEN Deficient Tumour Cells
Source: PLoS One. 2012 Oct 29;7(10):e47249. doi: 10.1371/journal.pone.0047249 (PMC3483146; doi:10.1371/journal.pone.0047249)
Supplement: Figure S2 — Evaluation of control siRNAs (siCON and siPLK1) viability effects in a panel of 24 tumour cell lines. “Deficient” and “Proficient” indicate PTEN status. (PDF) [file pone.0047249.s002.pdf]

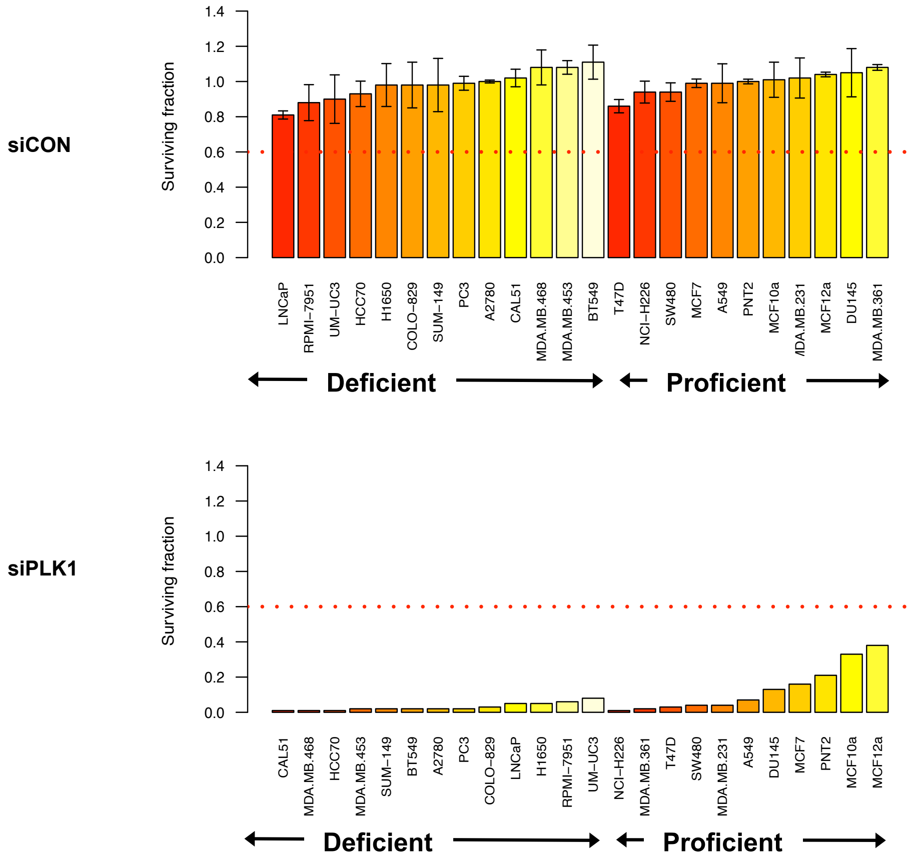

Figure S2. Evaluation of control siRNAs (siCON and siPLK1) viability effects in a panel of 24 tumour cell lines. “Deficient” and “Proficient” indicate PTEN status.
