# Supplementary material for: NLK Is a Novel Therapeutic Target for PTEN Deficient Tumour Cells
Source: PLoS One. 2012 Oct 29;7(10):e47249. doi: 10.1371/journal.pone.0047249 (PMC3483146; doi:10.1371/journal.pone.0047249)
Supplement: Figure S3 — Confirmation of silencing by FOXO1 and SKP2 siRNAs in HCT116 cells. Antibodies targeting epitopes of FOXO1 (C29H4, cell signaling #2880) and SKP2 (cell signaling #4358) were used for immuno-blotting. (PDF) [file pone.0047249.s003.pdf]

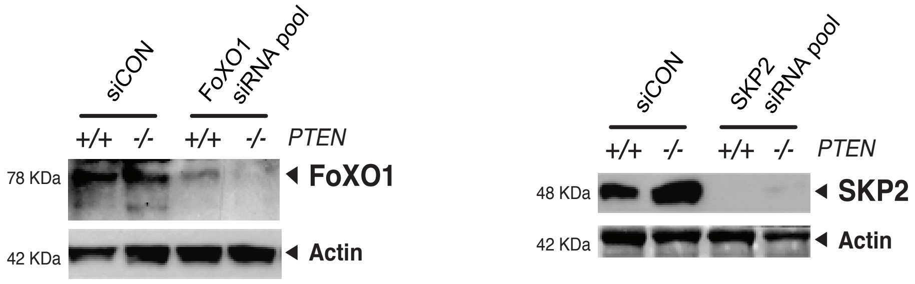

Figure S3. Confirmation of silencing by FOXO1 and SKP2 siRNAs in HCT116 cells. Antibodies targeting epitopes of FOXO1 (C29H4, cell signaling #2880) and SKP2 (cell signaling #4358) were used for immuno-blotting.
