# Supplementary material for: NLK Is a Novel Therapeutic Target for PTEN Deficient Tumour Cells
Source: PLoS One. 2012 Oct 29;7(10):e47249. doi: 10.1371/journal.pone.0047249 (PMC3483146; doi:10.1371/journal.pone.0047249)
Supplement: Table S1 — List of synthetic candidate hits from siKinome isogenic screen highlighting Nemo-like kinase (NLK) results. (PDF) [file pone.0047249.s004.pdf]

GeneID	DeltaZ	koNPI	wtNPI	DeltaNPI
SCAP1	-1.66	0.79	1.15	-0.36
NLK	-1.72	0.69	0.99	-0.30
BRDT	-1.80	0.34	0.64	-0.30
TESK2	-1.28	0.64	0.93	-0.29
CDC2L2	-1.43	0.37	0.64	-0.27
CDKL3	-1.55	0.80	1.06	-0.26
LMTK3	-2.05	0.41	0.65	-0.24
TLR3	-1.00	0.29	0.53	-0.24
EPHB2	-1.20	0.77	1.01	-0.24
SNF1LK	-1.46	0.70	0.92	-0.22
SPHK2	*-0.96*	*0.27*	*0.49*	*-0.22*
PLK4	-0.93	0.62	0.84	-0.22
TBK1	-0.94	0.73	0.95	-0.22
TNFRSF10A	*-0.87*	*0.29*	*0.50*	*-0.21*
SEPHS2	-0.79	0.65	0.86	-0.21
PTK2B	-1.41	0.70	0.90	-0.20
TAF1	*-1.12*	*0.22*	*0.42*	*-0.20*
LYK5	-0.98	0.91	1.10	-0.19
DLG3	*-2.05*	*0.11*	*0.30*	*-0.19*
TESK1	-0.82	0.33	0.52	-0.19
TTK	-0.93	0.32	0.51	-0.19

Table S1. List of synthetic candidate hits from siKinome isogenic screen highlighting Nemo-like kinase (NLK) results.
